# Adipose-Derived Stem Cell-Incubated HA-Rich Sponge Matrix Implant Modulates Oxidative Stress to Enhance VEGF and TGF-*β* Secretions for Extracellular Matrix Reconstruction *In Vivo*

**DOI:** 10.1155/2022/9355692

**Published:** 2022-01-17

**Authors:** Yu-Shen Cheng, Hung-Hsun Yen, Chung-Yen Chang, Wei-Chih Lien, Shu-Hung Huang, Su-Shin Lee, Lin Wang, Hui-Min David Wang

**Affiliations:** ^1^Department of Chemical and Materials Engineering, National Yunlin University of Science and Technology, Douliou, Yunlin 64002, Taiwan; ^2^Department of Fragrance and Cosmetic Science, Kaohsiung Medical University, Kaohsiung 807, Taiwan; ^3^Department of Physical Medicine and Rehabilitation, National Cheng Kung University Hospital, College of Medicine, National Cheng Kung University, Tainan 701, Taiwan; ^4^Ph.D. Program in Tissue Engineering and Regenerative Medicine, National Chung Hsing University, Taichung 402, Taiwan; ^5^Division of Plastic Surgery, Department of Surgery, Kaohsiung Medical University Hospital, Kaohsiung 807, Taiwan; ^6^Collage of Medicine, Kaohsiung Medical University, Kaohsiung 807, Taiwan; ^7^College of Chemistry & Pharmacy, Northwest A&F University, Yangling, Shaanxi 712100, China; ^8^Graduate Institute of Biomedical Engineering, National Chung Hsing University, Taichung 402, Taiwan; ^9^Department of Medical Laboratory Science and Biotechnology, China Medical University, Taichung 404, Taiwan

## Abstract

This study demonstrated both adipose-derived stem cells (ASCs) *in vitro* and *in vivo* combined with three-dimensional (3D) porous sponge matrices on implant wound healing. Sponge matrices were created from hyaluronic acid (HA), collagen (Col), and gelatin (Gel), constructing two types: HA-L (low content) and HA-H (high content), to be cross-linked with 1-ethyl-3-(3-dimethylaminopropyl) carbodiimide (EDC). Fourier transform infrared spectroscopy method verified carboxyl groups of HA and amino groups of Col and Gel reacting between the raw materials and scaffolds to identify the successive cross-linking. The swelling ratios of two types of sponge matrices were analyzed by water absorption capabilities, and the results displayed both over 30-fold dry scaffold weight enhancements. In biodegradation tests, matrices were hydrolyzed over time by three cutaneous enzymes, hyaluronidase, lysozyme, and collagenase I. ASCs from rats were cultured within the HA-H scaffold, demonstrating higher antioxidative abilities and secretions on related genes and proteins compared to the other two groups. The ASC HA-H matrix promoted cell proliferation to stimulate capillary angiogenesis inducer secretions, including vascular endothelial growth factor (VEGF) and transforming growth factor-*β* (TGF-*β*). *In vivo* histological examinations showed ASCs from implanted HA-H implant transported into the subcutis, and rat skin cells also infiltrated into the original matrix zone to increase the extracellular matrix (ECM) reconstructions. Our experimental data revealed that the ASC HA-H sponge implant was effective in improving wound repair.

## 1. Introduction

The human skin covers the entire body, has an average surface area of nearly 1.6 m^2^ in a human adult, and acts as the first line of defense against external factors. The skin comprises three layers: epidermis, dermis, and subcutis [1]. During cell differentiation, the epidermis divides into the stratum corneum, granulosum, and spinosum and basal layer [2]. The basal layer consists of basal cells, which act as daughter cells, continuously dividing and moving toward the outer layer of the skin. The dermis area is the supportive connective tissue between the epidermis and the underlying hypodermis and includes two layers: the dermis papillary and reticular layers. The human dermis contains sweat glands, hair roots, nervous cells, fibers, and blood capillaries and lymph vessels. The subcutis, also called the hypodermis, lies below the dermis and is used for fat storage. It contains loose connective tissues and fat lobules and has larger blood vessels and nerves than the dermis [2].

Stem cells are biological undifferentiated cells which differentiate to specialized ones and divide into producing more stem cells and are found within multiple organism tissues [2, 3]. In mammal organisms, stem cells comprise two broad kinds: embryonic and adult stem cells, whereby the former are found in blastocyst inner cell mass and the latter are purified from various tissues [4]. In adult creatures, progenitor and stem cells work as the healing medical staffs to the body, supporting matured organs. There are three identified available resources of autologous adult stem cells: (1) adipose tissue (lipid cell), which is required for extraction by liposuction; (2) bone marrow, which is needed for extraction by harvest, that is, drilling into bone (typically the femur or iliac crest); and (3) blood, which is necessitated for extraction through apheresis, wherein blood is drawn from the donor (similar to a blood donation) and passes through a machine that extracts the stem cells and returns other portions of the blood to the donor [5]. Adipose-derived stem cells (ASCs) ideally possess other unique properties, including abundant proliferation, minimally invasive procedures for collections, and easy adaptation when transplanted into a host [6, 7]. ASCs can be isolated from a plentiful source of adipose tissues recovered through lipoaspirations, which have become a routine plastic procedure with the increasing prevalence of obesity and the extra lipid accumulation [7]. These subcutaneous adipose tissues greatly contribute to research and other medical applications. ASCs have presented to own the DNA regenerative defective ability, having crucial positive impacts on extensively damaged oxidative stress cells or tissues [8]. It was identified that reactive oxygen species (ROS) improved stem cell differentiations and assisted reprogramming them into induced pluripotent stem cells (iPSCs), and on the other hand, ROS were related to the premature aging and malignant cell transformation. High oxidative stress hinders wound repair and slows the healing process. Since ASCs were discovered, several summary points have been revealed on their differentiations and wound-healings and tissue-engineering capacities [7–10]. We also have published several papers to demonstrate this finding [3, 11–14].

Designing specific therapeutic properties can help to decrease the morbidity and mortality from full-thickness skin wounds by using skin substitutes [15]. Nowadays, tissue engineering approaches apply three-dimensional (3D) skin substitutes including tissue cells, scaffold matrices, and surrounding signals, to accomplish this goal. In the past few decades, various potential scaffold matrices were developed from synthetic and natural polymers for tissue repairs and regenerations, according to the principles of tissue engineering [16, 17]. 3D porous matrices serve as temporary templates to guide new tissue reproduction and provide sufficient porosity for body fluids to transport nutrients and oxygen [18]. Biomaterials, including hyaluronic acid (HA), collagen (Col), and gelatin (Gel), which are the major components of human connective tissues, soft tissues, and the skin, ideally serve as physical support edifices of the extracellular matrix (ECM) [19, 20]. The present study hypothesized that HA, Col, and Gel could be cross-linked together into a 3D porous matrix and used as a cultured construction for ASC proliferations. This ASC matrix implant was assessed for biocompatibilities and characteristics *in vitro*, ex vivo, and *in vivo* for skin wound repair. We found out this implant was positively and potentially applied in wound healing.

## 2. Materials and Methods

### 2.1. Preparation of Porous Matrices

HA (molecular weight: 2–2.1 million daltons; Kibun Food Chemicals Co., Japan), Col (molecular weight: 64,000 daltons; Sigma-Aldrich Co., St. Louis, MO, USA), and Gel (molecular weight: 5,000 daltons; Sigma-Aldrich Co.) were initially dissolved into 100.0 mL deionized water. The raw material ratio for HA-L (low HA content) was HA : Col : Gel =2.43 : 36.58 : 60.99 (g/g, %), and another for HA-H (high HA content) was 38.46 : 23.08 : 38.46 (g/g, %). Both homogeneously mixed solutions were dispensed at −80°C into a specific dish, which had the same curvature as contact lenses. The frozen mixtures were lyophilized in a freeze dryer (FDU-1200, Eyela Co., Japan). 1-Ethyl-3-(3-dimethylaminopropyl) carbodiimide (EDC, Sigma-Aldrich Co.) was then added to the mixture and thoroughly mixed at 4°C to form a solution with a mass ratio of EDC : mixture of 1 : 12, and the cross-linking reaction solution was stirred for 24 h. After the cross-linking was completed, the films were rinsed thrice with deionized water and lyophilized again. The freeze dryer was under harsh conditions for sterilization and HA-L dry weight of 5.22 ± 0.01 g and HA-H dry weight of 5.30 ± 0.01 g.

### 2.2. *In Vitro* Matrix Characterization: Fourier-Transform Infrared Spectroscopy (FTIR) Scanning

FTIR is a type of light signal applied to acquire the infrared spectrum of absorption or emission of gas, liquid, or solid materials. FTIR spectrometer 2000 system (PerkinElmer, OH, USA) was used for the simultaneous collections of high-spectral-resolution data over wide spectral ranges. A matrix sample was made into a slight powder, mixed with KBr powder (1 : 8), dried in a 70°C oven for 24 h, and compressed into pellets for FTIR examination over a wavenumber range between 400 and 4,000 cm^−1^ with a scanning speed of 2.5 cm/s and a resolution of 2 cm^−1^.

### 2.3. *In Vitro* Matrix Characterization: Swelling Ratio

The swelling ratios of matrices were determined by a conventional gravimetric procedure [21] and were performed on samples immersed in phosphate-buffered saline (PBS) at pH 7.4 using a thermostatic water bath at 25°C. Samples were made of similar sizes for the examinations (approximately 10.0 mm in diameter in circle shape and 2.0–3.0 mm in thickness). The weights of the swollen matrix samples (*W*_0_) were determined immediately after removing excess water with tissue paper. The equilibrium weight of the swollen gel samples (*W*_1_) was obtained until no measurable weight increase was observed. The swelling ratio was calculated as follows:

(1) $$\begin{array}{c} \text{Swelling ratio}=\frac{W_{1}-W_{0}}{W_{0}}\;\left(\text{fold}\right). \end{array}$$

### 2.4. *In Vitro* Matrix Characterization: Enzymatic Degradations

Three enzymes, hyaluronidase, lysozyme, and collagenase I (Sigma-Aldrich Co.), were used for the degradation of two types of matrices. Hyaluronidase and lysozyme are important enzymatic proteins of the ECM metabolism which catalyzes the degradation of the dermal ECM surrounding glycosaminoglycans [22]. They hydrolyze glycol bonds of HA 1,4-*β*-linkages between *N*-acetyl-*D*-glucosamine residues and *N*-acetylmuramic acid. Collagenase I specifically targets the relatively loose region (Gly775-Leu/Ile776) of the Col triple helix domain. Waterless matrices (30.0 ± 0.03 mg) were absorbed in 1 mL PBS buffer (pH 7.4) with hyaluronidase (30 and 50 U/mL) for 4-, 8-, and 12-day incubations, to examine two matrix degradation rates. A similar protocol was used for lysozyme (10,000 and 30,000 U/mL) degradations for 2, 4, and 6 days. In collagenase I biodegradation tests, 10.0 and 20.0 U/mL collagenase I were suspended in 1.0 mL 0.1 M Tris–HCl with 0.05 M CaCl_2_ (pH 7.4), and matrices were immersed for 4, 8, and 12 h. All reactions were incubated at 37°C, and 0.2 mL of 0.25 M ethylenediaminetetraacetic acid (EDTA, Sigma-Aldrich Co.) was added to terminate the reactions at the specified time points. At the end of the evaluation periods, the remaining matrices were washed three times in distilled water and finally lyophilized for the final weight amounts. The degradation ratio was calculated as the percentage via dividing the difference between the residual weight (*W*_*n*_) and original weight (*W*_0_) by the original weight (*W*_0_):

(2) $$\begin{array}{c} \text{Degration ratio}\left(\%\right)=\frac{W_{n}-W_{0}}{W_{0}}\times 100\%. \end{array}$$

### 2.5. Collections of Adipose Tissues from Sprague–Dawley (SD) Rats

Adipose tissues were harvested from six male SD rats (BioLASCO Taiwan Co., Ltd), which weighed approximately 300–400 g. The rats were first anesthetized by intraperitoneally injecting Zoletil 50. Their inguinal areas were then cut out to collect approximately 10 g of adipose tissue. The Institutional Animal Care and Use Committee at National Chung Hsing University approved the described procedures (NCHU-IACUC No. 106–123). Since the collected adipose tissues still contained erythrocytes, Dulbecco PBS (D-PBS) buffer was used to rinse the adipose tissues and remove most of the erythrocytes. The tissues were scraped into a tube, washed with D-PBS buffer, and centrifuged at 750 × g for 5 min, similar to the protocol described by Bergonzi et al. [23]. The upper layer was then transferred into a new test tube using a sterile pipette and was washed at least twice. A second centrifugation was performed (750 × g for 5 min), and the tissues were transferred into tubes containing Dulbecco's modified Eagle medium (DMEM, Thermo Fisher Scientific Co., Waltham, MA, USA) with 1 mg/mL collagenase, 0.2 mM ascorbic acid 2-phosphate, and 2 mM n-acetylcysteine (NAC). The tubes were submerged for 3 h at 37°C and centrifuged at 750 × g for 5 min to separate the collagenase solution. The precipitate was washed following the centrifugation and cultured for 24 h into DMEM with 10% fetal bovine serum (FBS), 2.0 mM NAC, and 0.2 mM ascorbic acid 2-phosphate in a 5% CO_2_ incubator. Any unattached cells were removed using PBS. 25 cm^2^ flasks were prepared, each containing 5.0 mL keratinocyte serum-free medium (SFM) with 10% FBS, 100 U/mL penicillin, and 100.0 mg/mL streptomycin. The medium was replaced every other day. Cells were prepared for subculture, and any excess was stored in a liquid nitrogen tank.

### 2.6. Fluorescence Microscopy Image of Ex Vivo ASC Matrix

After a centrifugation, cell pellets (2 × 10^5^ cells/mL) were mixed with 5 mL PKH26 (Sigma-Aldrich Co.), a red fluorescent dye that was stained for cell membrane labeling. The mixtures were then sunk for 5 min at 25°C according to the experimental protocol and were vortexed for 30 sec. Excess PKH26 was washed with a complete medium. A monolayer of PKH26-labeled ASCs was reseeded into two types of matrices at a density of 1 × 10^5^ cells/mL. PKH26-labeled cells from both matrices were harvested in a Cell Proliferation Kit II [2,3-bis(2-methoxy-4-nitro-5-sulfophenyl)-2H-tetrazolium-5-carboxanilide, XTT; Life Technologies, CA, USA] assay for the cell proliferation measurement and viewed using a fluorescence microscopy (Eclipse Ti-U; Nikon, Japan) for the cell morphology [3].

### 2.7. Measurement of Intracellular ROS Level in ADSC

ROS-sensitive fluorescent dye, 2′,7′-dichlorofluorescin diacetate (DCFDA) was applied to determine if the HA-rich sponge matrix downregulated the intracellular oxidative stress level in cells [24, 25]. In order to observe the sample antioxidative properties, cells were precultured within two types of biomaterials for 24 hrs. Afterward, it was rinsed with warm PBS solution and incubated within 20 *μ*M DCFDA-contained PBS at 37°C and 5% CO_2_ for 30 min, to replace a fresh cell medium and to wash cells at least 3 times with PBS. Using trypsin/EDTA, we cut away the focal adhesion cell anchored to the culture dish. The cellular fluorescent intensity was analyzed with Guava® easyCyte Flow Cytometers (Merck KGaA, Darmstadt, Germany) at 485 nm excitation and 530 nm emission for 2,7-dichlorofluorescein (DCF) detection.

### 2.8. Scanning Electron Microscopy (SEM) Image of Ex Vivo ASC Matrix

SEM is a technique of electron microscope applied to acquire images of a sample via scanning the surface through a focused electron beam. The electrons interact within sample atoms to produce multiple signals which contain the information about the sample compositions and the surface topography. The morphological characteristics of the ASC (5 × 10^3^ cells/mL) HA-H porous matrix implants were observed using a SEM (JSM-5300; JEOL Co., MA, USA) [3]. Cells were cultured in keratinocyte-SFM with 10% FBS, 100 U/mL penicillin, and 100 mg/mL streptomycin in a 5% CO^2^ incubator. Matrices were fixed in 2.5% glutaraldehyde in 0.1 M sodium phosphate buffer (pH 7.2) overnight, postfixed in 1% osmium tetroxide for 1 h, dehydrated in ethanol, and dried at the critical point. Dried samples were coated with gold using a sputter coater (JEE-4X/5B; JEOL) at an ambient temperature. We took images with a cell-free matrix and ASC HA-H porous matrix implant cultured for 14 and 42 days in suitable view scales.

### 2.9. Examination of Ex Vivo Cell Proliferation Ratio

The principle of XTT is similar to MTT, and the final product of XTT is water-soluble. In our previous research [3], we found that XTT examination on the proliferation rate in the scaffold was more accurate than the MTT assay. An aliquot of the cell suspension (5 × 10^3^ cells/mL) was injected into each matrix. Cells were cultured as in previous procedures. On days 1, 3, 5, and 7, the XTT assay was applied to detect cell proliferation. This platform detects one of the mitochondrial functions, i.e., the reduction of XTT into a formazan product. Each well received 400 *μ*L XTT medium, and the plate was dipped for 2 h. A microplate reader (Stat Fax, Palm City, FL, USA) was applied to measure cellular XTT optical density at 450 nm. The percentage of cell proliferation was calculated by subtracting the absorbance of the blank from the total absorbance (*A*) on each time interval. The result was then divided by the difference between the absorbance on day 1 (*A*_1_) and that of the blank as shown:

(3) $$\begin{array}{c} \text{Percentage of cell proliferation}=\frac{A-A_{\text{blank}}}{A_{1}-A_{\text{blank}}}\times 100\%. \end{array}$$

### 2.10. Implanting ASC HA-H Matrix in Animals

According to the ex vivo cell proliferation ratio protocol, 4 × 10^5^ cells/mL were seeded in a 2 × 3 cm^2^ matrix. Before seeding, ASCs were stained with PKH26 for histology and immunofluorescence detection [14]. The HA-H matrix only and ASC HA-H matrix were implanted in the back of adult male SD rats weighing 320 ± 20 g. SD rats were housed in an animal facility at 22°C and a relative humidity of 55% with a 12 h light–dark cycle. Food and sterile tap water were available ad libitum. All procedures were approved by NCHU-IACUC No. 106-123. The dorsum subcutis of SD rats was implanted with two types of ASC-cultured matrices under anesthesia with 2.0%–2.5% isoflurane (Halocarbon Products Co., GA, USA) in O_2_.

### 2.11. Histological Detection of ASC HA-H Matrix Implant by Hematoxylin and Eosin (H&E) and Immunohistochemistry Staining

4′,6-Diamidino-2-phenylindole (DAPI, AAT Bioquest Co., CA, USA) is a fluorescent stain which binds powerfully to double-strand DNA in adenine–thymine-rich regions. DAPI is a well-known nuclear counter mark to apply in multicolor fluorescent techniques and following the protocols of Sigma-Aldrich Co. to stain skin tissue cells. ASCs were stained with PKH26 and then seeded in a matrix. SD rats were sacrificed in seven days after the implant surgery by an overdose of CO_2_. The ASC implant dorsum and normal skins were separated for histological examination using H&E staining and for fluorescent observations [26]. The section used for H&E staining was formalin-fixed and embedded in paraffin; 10 *μ*m thick sections were cut and mounted on glass slides. The sections were deparaffinized in xylene and stained with H&E. For fluorescence observations, an optimal cutting temperature compound (OCT; Finetek Co., CA, USA) was used to bind the tissue at −80°C. The frozen sections were cut into 10 *μ*m thick slices by a Shandon cryotome (FSE, Thermo Fisher Scientific Co.), rinsed three times with PBS buffer for 5 min, and mounted with FluoroQuest™ Mounting Medium containing DAPI. The same procedures were used for microscopy, as described in the previous paragraph for fluorescence microscopy of *in vivo* tissue matrix.

### 2.12. Quantitative Real-Time Polymerase Chain Reaction (qRT-PCR)

The qRT-PCR method is a technique used for evaluating gene expression levels by measuring the cDNA products after each cycle of PCR amplifications. For qRT-PCR, a reactive mixture containing SYBR Green Master Mix (Qiagen, Valencia, CA, USA) templates and primers was used. All qRT-PCR reactions were accomplished by a StepOnePlus™ System (Thermo Fisher Scientific Co.). The reactions were carried out according to the following program: cDNA templates were initiated at 95°C, annealed at 60°C, and elongated at 72°C, and all steps were repeated with 40 cycles of amplifications. At the end of the annealing stage of the experiments, we began to determine the fluorescence acquisition [27]. The targeted gene primers are shown in Table S1.

### 2.13. Western Blot Analysis

Western blot is a famous method to separate proteins and to identify them, and the sample sources are from the cell cultures or sacrificed tissues. Specific binding antibodies for corresponding proteins were fitted to analyze the performances of the proteins in our rat tissues. Western blot analysis is the primary method for transferring proteins, which is termed electroblotting. Lysates were centrifuged at 20,000 × g for 30 min, and the protein concentrations within the supernatant solution were determined with a bicinchoninic acid (BCA) protein assay kit (Pierce Co., Rockford, IL, USA). Equal amounts of protein were separated by sodium dodecyl sulfate-polyacrylamide gel electrophoresis (SDS-PAGE) and then electrotransferred to a nitrocellulose polyvinylidene difluoride (PVDF) membrane (PALL Life Science, Ann Arbor, MI, USA) using an electrical current. Then, the membrane was washed with 1x TBST (Tris-buffered saline, 0.1% Tween 20) to remove traces of skim milk and placed in the TBST box. The membrane was immersed with respective primary antibodies and incubated with the horseradish peroxidase-conjugated second antibodies for 1 h and then treated with enhanced chemiluminescence (ECL) detection reagents (PerkinElmer, ECL1 : ECL2 = 1 : 1, Spokane, WA, USA). The protein signals after ECL treatments were visualized by a Mini Size Chemiluminescent Imaging System from Life Sciences (Thermo Fisher Scientific Co.) and were measured to detect the bands [14].

### 2.14. *In Vivo* Secretions of Angiogenic Growth Factors by ASC HA-H Matrix Implant

Sacrificed tissues were collected to detect secretions of proangiogenic cytokines on days 1, 3, 5, and 7 [14]. According to the manufacturer's instructions, we made an enzyme-linked immunosorbent assay (ELISA) to determine the amounts of vascular endothelial growth factor (VEGF) and transforming growth factor-*β* (TGF-*β*) secreted by two ASC-implanted tissues following ELISA development kits (BlueGene Biotech Co., Shanghai, China). Briefly, 10 mg skin tissue was collected from rats, cut into pieces, and suspended in lysis buffer overnight to extract protein. 100 *μ*L samples were added into ELISA plates which coated the VEGF and TGF-*β* antibody and were incubated at room temperature for 2 h. After incubation, 100 *μ*L of Streptavidin-HRP Conjugate was added for 30 min and detection by spectrophotometer at 620 nm.

### 2.15. Statistical Analysis

Differences between the vehicle control group and ASC-treated matrix groups *in vitro*, ex vivo, and *in vivo* assays were analyzed by Student *t*-test. One-way ANOVA was applied for statistical comparisons between the vehicle control group and experimental groups. A significant difference (∗) was defined as *p* < 0.05, and a highly significant difference (∗∗) was defined as *p* < 0.01.

## 3. Results and Discussion

### 3.1. Chemical Cross-Linking Reaction for Extracellular Matrix-Like Porous Matrices

**Figure 1:**
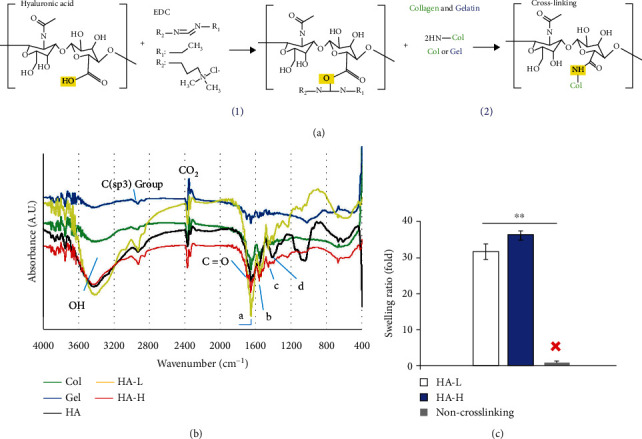
(a) The two-step chemical synthesis process for cross-linking matrices: (1) the cross-linking agent, EDC, linked HA with another organic molecule to form the activated intermediate acid anhydride. (2) The carboxyl groups of the activated intermediated product reacted with the amino group of Col or Gel to complete the cross-linking reaction. (b) FTIR spectra absorbance vs. wavenumber (cm^−1^) data: HA (black), Col (green), Gel (blue), HA-L (yellow), and HA-H (red); absorbance (A.U.) and wavenumber (cm^−1^). The molecules overlapped at ~1700 cm^−1^, indicating an amide bond for the C=O stretching vibration. (c) The swelling ratio of testing samples: HA-L/HA-H/non-cross-linking. The white and blue bars represented HA-L and HA-H implant matrices, respectively. Without cross-linking, the scaffold was not formed in the water phase solution. Each value was represented as the mean values ± standard deviation (SD) (*n* = 3). The level of significance was designated as ^∗^*p* < 0.05 and ^∗∗^*p* < 0.01.

HA, an anionic and nonsulfated high molecule glycosaminoglycan, is found in connective, epithelial, and neural tissues. It is one of the key components of wound repair, significantly contributing to the stimulation of initiation, release, and regulation of angiogenic growth factors [28]. HA not only plays a diversity of roles in pathologic and physiologic issues but also is employed extensively in clinical and skin cosmetic applications as a biomedical agent and drug delivery system supplement. The main restriction of HA is because of rapid degradation, short half-life property, and its accordingly low bioavailability in vivo. In our aim to conquer the above weaknesses, HA is typically subjected to chemical modifications [29]. Col is the major component of ECM and comprises approximately 70%–80% of dry skin weight [30]. As one of the elements, Col has many advantages, including biocompatibility, low toxicity, and nonimmunogenic properties. Gel is a partial hydrolysis product of Col and can be manufactured easily in an inexpensive approach. Gel is a common supplement to support biomaterial product physical and structural stability and is usually used in the chemical, food, and pharmaceutical industries. The construction of 3D matrices via cross-linking has become one of the main strategies for creating porous matrices [31]. We expected to cross-link these three components modifying the matrix degradation rates, water absorption, and biomechanical characteristics, thus further improving their biocompatible implant applications. In this study, EDC was used as a chemical cross-linking agent, which is a zero-length cross-linking agent (i.e., the agent itself is not incorporated into the macromolecule) [32], to directly connect to two different reactive groups demonstrating the chemical scheme in Figure 1(a). The activated intermediate agent reacted with the carboxyl groups of HA and with various amine side chains of Col and Gel and linked HA to Col and Gel forming a porous matrix. Using lyophilization, we could obtain a sponge-like matrix using the above method, and the following steps tested the characteristics of the porous matrix cellular in vitro, ex vivo, and in vivo.

### 3.2. Porous Matrix Characterization: FTIR Scanning

FTIR spectroscopy glowed a beam light containing multiple frequencies at once and evaluated the beam absorption by the material. Next, the beam light was modified containing a different frequency combination to give a second result point. This process was repeated multiple times, and then, a computer calculated all the results and worked backward to infer the absorption of every wavelength [3]. Through our preliminary studies, we found the enhancements of HA contents in the biomaterial assisted the ASC proliferations. However, the high viscosity was a big manufacturing problem while the HA concentration was augmented in the reaction solution. We applied two HA concentrations for our further implant examinations. FTIR spectra of the matrices along with their individual polymer components are shown in Figure 1(b). In FTIR spectra of all components, peaks at 1,655 and 1,560 cm^−1^ correspond to amide I for the C=O stretching vibration and amide II for the N–H bending vibration. The peak area at 1,455 cm^−1^ corresponds to the C–N bending. In the pure HA spectrum, peak areas at 1,042 and 1,080 cm^−1^ correspond to C–O–C and C–O stretching, respectively. The absorption peaks at 1,400 cm^−1^ corresponded to the symmetrical stretching of vibration bands of the carboxyl groups. The absorption peaks of the amide group in the matrix polymers highly overlap with those newly cross-linked by the EDC amide groups in the HA-L and HA-H matrices. Therefore, no prominent new absorption peaks were observed in the HA-L and HA-H spectra.

### 3.3. Porous Matrix Characterization: Swelling Ratios

The swelling of polymeric 3D materials is a mechanic fluid diffusion-interrelated process to contain the liquids. The swelling ratio measurement is a low-molecular solvent content within an elastomeric matrix network and is characteristically accounted as the proportion of either the volume or the mass of the matrix in the swollen status compared to that of the dry status [3]. Three types, HA-L matrix, HA-H matrix, and non-cross-linked raw materials, were tested for their abilities to absorb water using the swelling ratio assay. A high swelling ratio indicates a scaffold can transport nutrients and oxygen into the wound body around the zone. The swelling ratios are shown in Figure 1(c). The non-cross-linked materials were dissolved in water immediately (within 2 min) and could not even maintain their hydrogel type in the PBS buffer; therefore, the swelling ratio was zero. On the other hand, the cross-linked HA-L and HA-H matrices could absorb approximately 31.2 ± 2.4- and 36.5 ± 1.8-fold PBS buffer than the dry matrix masses, respectively. According to our experience, HA has a good water absorption, and the absorption value is difficult to increase after reaching a certain maximum plateau value. Therefore, to increase the absorption ability from 31.2-folds to 36.5-folds is a significant improvement.

### 3.4. Porous Matrix Characterization: Enzymatic Degradations

**Figure 2:**
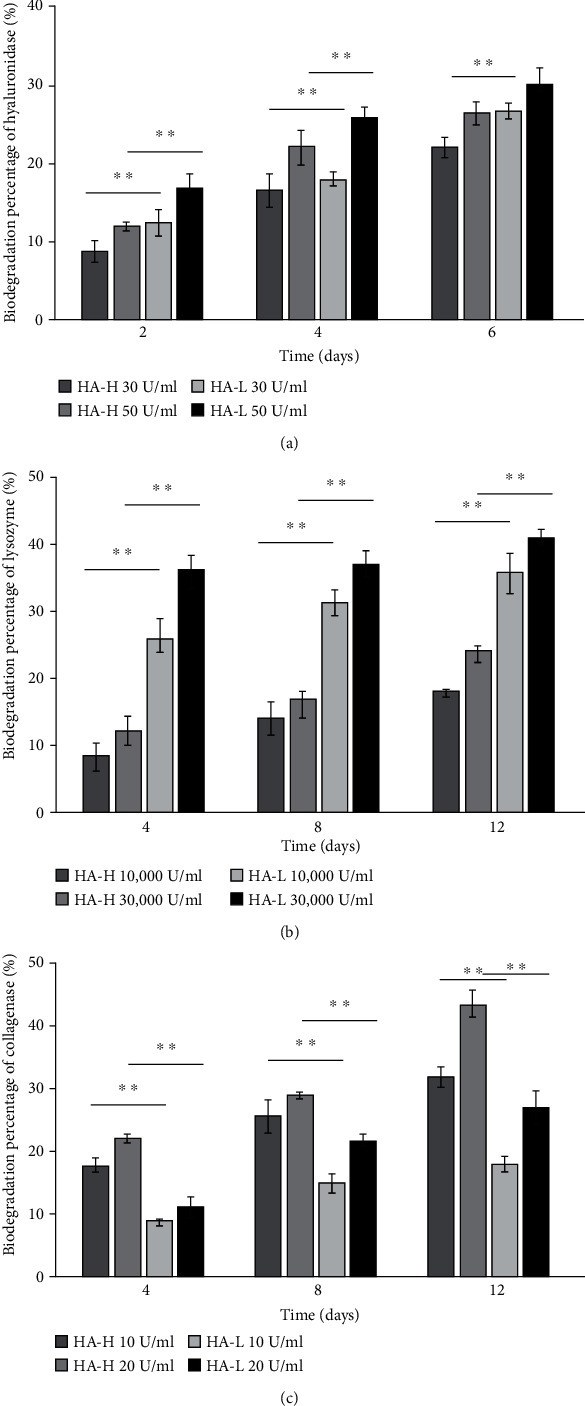
The matrix's HA glycoside was degraded by hyaluronidase and lysozyme, while the matrix's Col and Gel amino acid groups were degraded by collagenase. (a) Higher degradation percentages of hyaluronidase on HA-L matrix than on HA-H type. (b) Higher degradation percentages of lysozyme on HA-L matrix than on HA-H type. (c) Consistent increase of biodegradation percentages of collagenase in both type-tested matrices. Col and Gel molecular equivalent values were lower in HA-H matrix to make it easier to be hydrolyzed by collagenase I. Each value was represented as the mean values ± standard deviation (SD) (*n* = 3). The level of significance was designated as ^∗^*p* < 0.05 and ^∗∗^*p* < 0.01.

Therapeutic wound implants are temporary healing template structure materials, and biocompatible matrices need to be degraded eventually in the body. The degradation rate of a potent matrix should not be too high to ensure the sufficient time period is available for cells to proliferate and spread around the wound area. Besides, a matrix should not be a morphologically robust solid because it is not necessary to keep it in the individual's body permanently. Biological degradations of HA-H and HA-L matrices were determined by measuring a loss in the weight following three enzymatic degradations. The rates were assayed using in vitro hyaluronidase, lysozyme, and collagenase I, and these enzymes react with certain chemical functional groups of our matrix components. Hyaluronidase and lysozyme enzymatic proteins hydrolyze the HA glycol bonds. After six days of incubation, the percentages of biodegradation of HA-H and HA-L matrices with 30 U/mL hyaluronidase were 22.1 ± 1.3% and 26.5 ± 1.0%, and the values with 50 U/mL were 26.3 ± 1.5% and 29.8 ± 2.3% for two matrices, respectively, in Figure 2(a). The percentages of biodegradations in both matrices with 10,000 U/mL lysozyme were 18.2 ± 0.3% and 35.7 ± 3.0% after 12 days of the incubation time interval, and those with 30,000 U/mL were 24.3 ± 0.7% and 40.8 ± 1.3%, respectively, in Figure 2(b). Collagenase I cleaves the n-[3-(2-furyl)acryloyl]-L-leucyl-glycyl-L-prolyl-L-alanine (amino acid: FALGPA) groups of Col and Gel into FAL and GPA. After 12 h of reactions with 10 U/mL collagenase I, the biodegradation percentages of HA-H and HA-L matrices were 31.4 ± 1.5% and 17.4 ± 1.0%, and for two matrices with 20 U/mL for a half day, the hydrolysis ratios for two matrices were42.9 ± 2.5%and26.7 ± 2.7%, respectively, in Figure 2(c). Our data revealed two matrices were degraded in dosage- and time-dependent manners. In addition, a specific enzyme is just for the specific raw material component group, and it will be discussed in the following sections.

ECM plays an important role in supporting the base structure of new dermis and also assists fibroblasts in providing a framework of the base structure. Furthermore, ECM absorbs the growth factors and related protease of cells to help during wound repair periods [31]. HA was speculated to improve the secretion of ECM, which causes new dermis to be denser. The previous statement leads to ECM transcription, causing further regulation of differentiation into fibroblasts genes [32]. In different wound phases (inflammation, proliferation, and maturation), HA can play a role in inflammatory response moderation, reepithelialization, and scar tissue remodeling [33, 34]. HA is thought to be indispensable to decrease scars and is considered to reduce fibrosis during wound healing [35], and it is a main component in injury repair and a proangiogenic element in tissue engineering materials [36]. It reduces inflammatory reactions, oxidative stresses, and pain levels of wounds and increases vasculogenesis and angiogenesis for the regeneration [37, 38]. We take this antioxidative advantage to increase HA equivalent concentration normality within the 3D porous matrix implant improving the reduction of inflammation. Col plays a key role in each phase of the wound healing process, including proliferation, migration, and differentiation and is one of the most promising biological materials for such applications [39, 40]. In tissue engineering for wound dressing, Gel has many advantages, including being inexpensive and nonimmunogenic. Col, HA, and Gel generally possess well-controlled biodegradabilities and biocompatibilities, and to give a three-component physical matrix structure, we used EDC for the cross-link reaction to assist the hurt skin in would healing. As we know, a suitable biomaterial should contain both soft and tough properties in a suitable range to prevent it from being too stiff and damaging the tissues [40]. We found HA showed less affections on the hard mechanical and biological properties compared to the influences of Col and Gel. The excessive amounts of Col and Gel cause high rigidity of the porous matrix, and this is not good for wound repair [3]. HA ratio is an effective target raw material of ECM composition to augment pliability, and we raised the amount of HA material to slow the degradation rate in further studies. In addition, it is unsatisfying to see that the matrix degraded before the wound has not yet been repaired, due to the rapid completion of the biomaterial. HA is a degradable molecule; after the cross-link reaction with Col and Gel polymers, the sponge matrix has a long sustaining time issue. The water absorption property of HA is very well, but a limitation value exists which leads to similar water absorption amount between two materials. Despite the similar water absorption abilities of two scaffolds, HA-H showed better performance in other biochemical examinations including cell proliferations and VEGF expressions. We tried to enhance the concentration of HA up until the viscous limitation for the manufacturing operation. Skin cells secrete protease enzymes to degrade and digest matrices to generate sufficient space for cell proliferations and movement [8], and the matrices in this study were tested for their biological degradation. Hyaluronidase and lysozyme hydrolyze HA, whereas collagenase I degraded Col and Gel. In the enzymatic degradation reactions within examination time interval, a general pattern was shown: three specific enzymes degraded the glycol chemical bonds or FALGPA amino acid groups within two types of matrices, leading to similar results. We found out HA-L matrices had higher degradation rates with hyaluronidase and lysozyme than HA-H matrices because of the high HA contents with weak resistance to both enzymes [41]. Interestingly, collagenase I at high dosage showed the maximum matrix degradation percentage in the HA-H type, whereas collagenase I at slight concentrations presented the lowest value in the HA-L matrix. The HA-H matrix had a higher molecular equivalent in HA which is opposite to Col and Gel and with a lower resistance to collagenase I to digest Col and Gel. Hence, Col and Gel molecular equivalent values were lower in the HA-H matrix to make it easier be hydrolyzed by collagenase I. It was the reason why HA-L matrices were with lower biodegradations. Moreover, ECM contained certain cell growth factors to be as a regional warehouse; our HA-L matrix allowed the regional and speedy cellular growth factor-mediated active functions without resyntheses.

### 3.5. Ex Vivo ASCs Cultured in Matrices Evaluated by the Fluorescent Microscopy and Cell Proliferations

The cluster of differentiation (CD) marker is the biochemical description applied to the recognitions and research on the cellular surface supporting target biomolecules for the immunophenotyping [3]. In 2007, Schäffler and Büchler showed CD markers, differentiation capacities into various cell types, and clinical applications of ASCs [8]. In light of biochemistry and physiology, CD markers can work in many ways, frequently acting as peptide ligands or receptors essential to the physiological signals and functions. Currently, there are over 300 verified types of CD markers. Originally, CD90 was revealed as a thymocyte antigen of about 30 kDa with heavy N-glycosylated glycophosphatidylinositol conserved located on the cellular surface peptide, combining a single V-like immunoglobulin domain protein [42]. CD90 is utilized as one of the more popular indicators in a range of pluripotency stem cells, including ASCs. CD105 is a fragment of the TGF-*β* receptor complex, belonging to the cell surface type I membrane glycoprotein [43]. It is involved in angiogenesis and, consequently, is vital in stem cell proliferation and tumor metastasis in humans. CD146 is expressed in fetal and adult organ stem cells and is also recognized as the melanoma cell adhesion molecule anchored on the cellular surface in the body [44]. We identified the CD markers to verify where our rat ASCs belong, presented in the supplementary data in FigureS1.

In biochemical definitions, autologous stem cells are acquired from the human body, as someone may bank his or her own cells for medical elective therapies in the future, and among all category stem cell types, the autologous harvestry is in the lowest risky protocol for immune concerns [5]. Adult stem cells are often employed in surgical operations, such as in dermal repair and bone marrow transplantation. Scientists can manually grow, differentiate, and transform stem cells into particular cell types with hallmarks consistent with diverse tissues and organs, such as human skin and bones [45]. Embryonic stem cells and autologous cell lines obtained via somatic cell have also been proposed for positive therapies as future candidates [4]. An obvious interplay exists between the donor age and cell passage issues, which must be considered in developing cell-based therapies for future clinical applications. The data from diabetic rat wound healing research indicated stem cell injections, including bone marrow-derived mesenchymal stem cells (MSCs) and ASCs, resulting in faster wound repair [35]. ASCs prove an alternative origin of versatility stem cells to be differentiated into various tissue cells. ASCs have some advantages over other versatility stem cells, as seen in the following examples: adipose tissue is easy to be obtained, there is no immunological rejection of autologous transplanted cells, and it can be applied to a wide range of body tissues. A study on chronic diabetic foot ulcers included a 29-day treatment with an autologous graft comprising autologous skin fibroblasts combined with autologous stem cells on biodegradable Col membranes. The graft was directly applied to the wound and injected into the edges of the wound on days 1, 7, and 14. As a result, the injury size was decreased, and the vascularity of the dermis and dermal thickness of the wound were increased [46]. ASCs were chosen to be cocultured with our matrices because of their ability to regenerate and develop into specialized cells.

**Figure 3:**
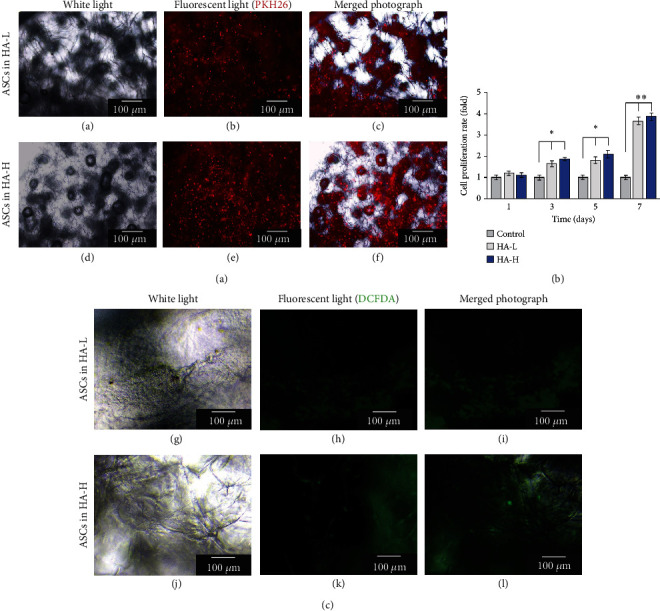
(a) View of cultured ASCs within two types of matrices under a fluorescence microscope. (A) and (D): The left column images were viewed under the white light. (B) and (E): The middle column images were viewed under the red fluorescence microscope. (C) and (F): The right column images were merged photographs of the first and second column ones (bar =100 *μ*m). (b) Steady enhancements of the cell proliferation rates for the vehicle control and HA-L and HA-H matrices. ASCs showed higher values cultured in HA-H matrix than in the HA-L one within a 7-day period culture. (c) The DCFDA assay results presented that HA-H matrix decreased ROS production in oxidative stress property assay. The photo representing order sequence (G)–(L) was similar as part demonstration (A)–(F). Each value was represented as the mean values ± SD (*n* = 3). The level of significance is designated as ^∗^*p* < 0.05 and ^∗∗^*p* < 0.01.

People frequently experience skin trauma suffering; thus, skin wound repair is highly significant in therapy medicine. To construct a tissue engineering healing unit which aids injuries is one of the approaches to help remedial processes, and matrices should be first tested for biocompatibility; i.e., they should not be harmful to normal cells and the body; otherwise, the patient's condition will be worsened. When a matrix is biocompatible, cells grow easily and fast, and the whole healing process is accelerated to recover. To observe the morphology of ASCs proliferating within the matrices, the cells were cultured in vitro in Petri dishes for 14 days. ASCs were stained with PKH26 and observed using a fluorescent microscope. The two rows, Figure 3(a), (A–C) and (D–F), show ASCs in HA-L and HA-H matrices photographed under white light and red fluorescence as well as the merged views (bar = 100 *μ*m). The matrices provided a 3D porous structure in which the porosity width was around 100–200 *μ*m for ASC migrations, and the microscope could only focus on one layer to observe ASCs in the matrices. All images showed ASCs with oval-shape morphologies. ASCs were seeded into two types of matrices, and the cell proliferation rates were examined using XTT assay on days 1, 3, 5, and 7, and the medium was changed every other day (Figure 3(b)). To compare with the experimental group on day 1, the cell viability showed enhancements between the control (onefold) and day 7 ASCs (>threefolds). Both types of matrix cell viabilities presented similar results in time-dependent manners, and HA-H groups showed slight augmentations compared to HA-L groups during the experimental periods. Antioxidative capacity is an important issue for wound healing because of the core function on reducing the radical-induced degradation and scavenging free radicals of repair cells and tissues. We already published several papers to demonstrate that oxidative lessening is beneficial to human health, especially in rejuvenation applications [47–49]. For measuring the antioxidative properties on the testing samples of interest, the cellular experimental platform was carried out in practice. DCFDA staining is a typical quantitative method for the intracellular H_2_O_2_ amounts to survey redundancy oxidation stresses. The damaged wound is a trigger for endogenous superoxide production and inflammatory activated cells formed ROS which release the fluorescent wavelength intensity to be identified [50–52]. To determine whether the HA-H matrix suppresses oxidative reactions, we investigate ROS generations in a cell-based measurement. The injured wound is an inducer for endogenous superoxide secretions, and inflammation-activated cells produced ROS which emitted the fluorescent intensity to be detected. In Figure 3(c), it was demonstrated that the HA-H matrix gradually reduced oxidative stresses, and we discovered that this biomaterial inhibited the production of cellular ROS successfully.

### 3.6. Ex Vivo ASCs Cultured in Matrices Evaluated by SEM

**Figure 4:**
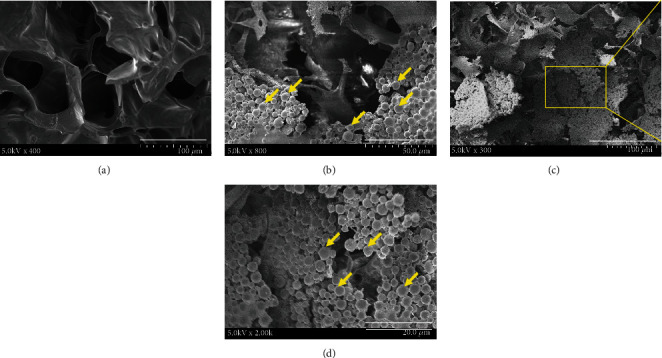
(a) SEM surface view of HA-H matrix only (without ASC culture) (bar = 100 *μ*m). (b) SEM surface view of HA-H matrix with ASC culture for 14 days (bar = 50 *μ*m). (c) SEM analysis of a 42-day ASC distribution on HA-H matrix (bar = 100 *μ*m) and (d) an enlarged lateral view (bar = 20 *μ*m).

For SEM measurements, a specimen is usually necessary to be entirely dry, since the specimen chamber is at a high vacuum condition, and the living cells, tissues, and organisms are required chemical fixations to stabilize and preserve the physiological structures. We aimed to construct a biocompatible matrix with a porous feature to help with nutrient and oxygen transportation from the entire body to the damaged area. ASC morphologies within HA-H matrix samples were also estimated using SEM. In Figure 4(a), a lateral view of HA-H matrix without ASC cultivation was shown, exhibiting interconnected open-pore morphology (bar = 100 *μ*m). We continually illustrated a lateral view of ASCs cultured in the HA-H matrix which is a longer period. In Figure 4(b), the matrix which was split after ASC fostering for 14 days was presented, demonstrating a good proliferative consequence (bar = 50 *μ*m). ASCs were widely dispersed in the HA-H matrix after 42 days of culture (Figures 4(c) and 4(d)). An enlarged view of the yellow-framed area was illustrated (bar = 20 *μ*m), and ASCs completely invaded the HA-H matrix.

During a 7-day period, cell proliferation rates in HA-L and HA-H matrices increased 3.5- and 4.2-folds compared with the original cell growths on day 1. The fluorescent examination images of the 14-day phenomena could identify ASC migrations and proliferations into two types of matrices. Next, ASCs proliferated for a longer time interval of 42 days to cover the surface of the HA-H matrix, and we discovered that ASCs migrated completely to the superficial around the matrix in the SEM images. Many types of cells bind to ECM components via focal adhesions connecting with actin filaments of the cell and via hemidesmosomes connecting with intermediate keratin filaments. Cellular adhesion is a mechanism for cell to attach and to interact with nearby cells by cell-adhesion molecules on the surface and cellular transmembrane proteins. In SEM, the fixed ASC tissue matrix was dehydrated to be detected. The air-drying protocol made our sample shrink and collapse, and we replaced the aqueous medium within the cells by the organic solvent of acetone achieving the real physiological structure discoveries.

### 3.7. In Vivo Histological Examination of Implanted ASC HA-H Matrix

**Figure 5:**
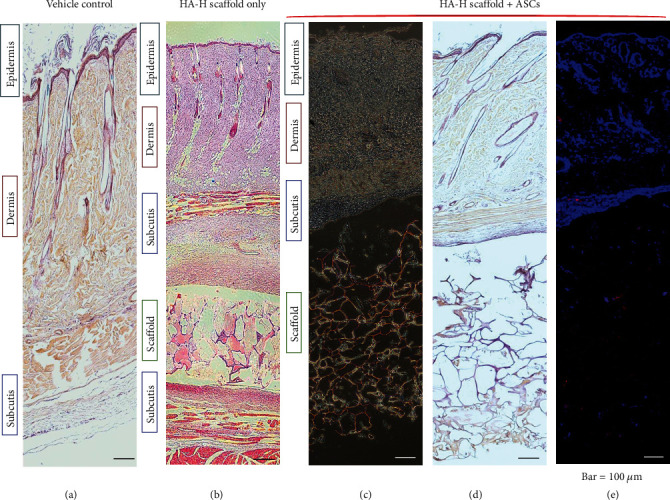
Histology images of wound beds. (a) Normal rat skin with paraffin section; (b) HA-H matrix was implanted into the rat skin. (c–e) ASC HA-H porous matrix was implanted within the rat skin. (c) Viewed with white light, (d) histochemical staining with the paraffin section, and (e) histochemical staining with the frozen section in fluorescent light. ASCs were dyed in PKH26 with a red color, and the blue color was a matrix surrounding tissue cells with DAPI staining.

The histological study of a tissue specimen is sectioned, stained, and examined by a light microscopy to observe the formations, structures, and functions. Our histological examinations of the rat skin and ASC matrix implant were performed by tissue sections. The goal was to evaluate skin histology with the implant of ASC HA-H matrix biocompatibilities and repair consequences. In Figures 5(a) and 5(b), both demonstrated the normal skin structure and a tissue section with the matrix implant only, respectively, and H&E staining revealed the matrix's and skin's histological characteristics (bar = 100 *μ*m). In Figures 5(c)–5(e), ASCs were stained with PKH26, and ASC HA-H matrices were sewn within the rat skin dermis for 7 days. It was shown that the skin accepted the ASC HA-H implant, and ASCs not only existed within the implant matrix but also migrated from the matrix into the subcutis-emitted red fluorescent light in Figure 5(e). Our ASC HA-H matrix implant provided ECM dynamic behaviors to regulate the cell functions, including segregating tissues from one another, supporting cells, and regulating intercellular communications. To compare the normal skin biopsy with the implanted skin, there is no significant or obvious dissimilarity between these histological sections. This suggested that the ASC matrix implant did not induce skin immune responses in vivo. We found that the matrix could be integrated with dermal multiple layers, and ASCs infiltrated into surrounding tissues. There was no significant difference between the normal skin and the ASC HA-H implant on histological examinations.

### 3.8. The Gene and Protein Expression Levels of Both ASC Biomaterial Implants

**Figure 6:**
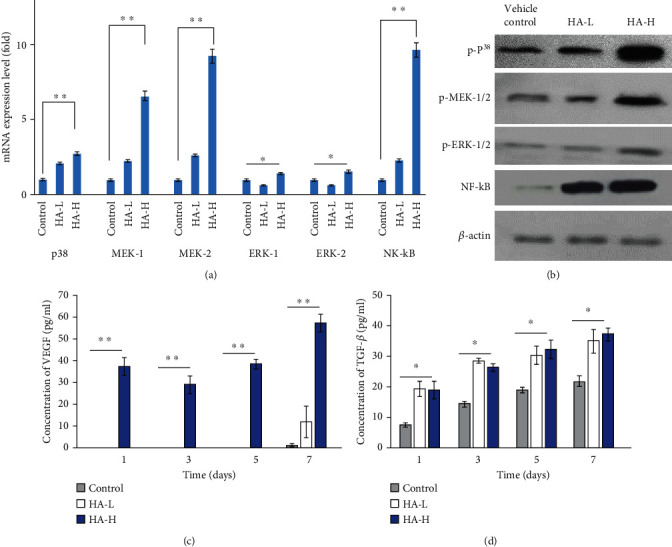
(a) The wound repair gene expression levels of ASCs from the vehicle control and both matrix implants in vivo. (b) The related protein expression amounts in the dish (vehicle control) and two types of implants. (c) VEGF-secreted concentrations (pg/mL) and (d) TGF-*β* secretions (pg/mL) of the vehicle control and two implants tested over a 7-day period. Each value was represented as the mean values ± SD (*n* = 3). The level of significance was designated as ^∗^*p* < 0.05 and ^∗∗^*p* < 0.01.

mRNA expression levels of two rat skin-implanted ASC matrices and the vehicle control via qRT-PCR are shown in Figure 6(a). There are three popular downstream wound healing network signals of mitogen-activated protein kinases (MAPKs): p38 mitogen-activated protein kinase (p38), mitogen-activated protein kinase kinase (MEK), and extracellular signal-regulated kinase (ERK). p38, MEK-1/2, ERK-1/2, and NF-*κ*B were at higher expression intensities of ASC implant tissues in the HA-H type than in the vehicle control group and HA-L one. These genes were positively related to wound repair, and MEK-1/2 and NF-*κ*B presented strong dramatic increase values more than fivefolds [53]. In Figure 6(b), we illustrated protein expression amounts of the rat skin-implanted ASC matrix and the vehicle control one in western blot. It was shown that p38, MEK-1/2, and NF-*κ*B were at higher expression quantities in the HA-H group compared with the vehicle control and HA-L groups (Figure S2). We presented a consilience result in both mRNA and protein expressions on our ASC HA-H matrix implant in vivo.

ECM-related genes and proteins are generally applied in cellular culture systems to preserve precursor and stem cells in an undifferentiated status in vitro during the induction of differentiated smooth muscle, endothelial, epithelial, and other cell types [38]. In molecular biology, p38 is responsive and activates via stress factors including ultraviolet irradiation, osmotic pressure, heat shock, and inflammatory cytokines, which are related to cellular growth, proliferation, angiogenesis, autophagy, and apoptosis. MEK is one of the MAPK signaling proteins and is also activated in melanoma [54]. If MEK is suppressed, the cellular differentiation is blocked, and the cell programmed death is induced. ERKs are broadly expressed protein kinase and intracellular signaling biomolecules related to cell activities, such as the regulation of mitosis and meiosis functions in differentiated cells. There are two similar protein kinases called ERK-1 and ERK-2, and the phosphorylation of ERKs leads to the activation of the kinase activities. The protein product of the protooncogene enhances p38 activity and therefore causes markedly high activity of the transcription factor NF-*κ*B [46]. NF-*κ*B is usually adjusted from intracellular pathways which integrate signals from the immune system and the neighboring tissues. Tyrosine receptor-linked kinases, p38, MEK, ERK, Ras, and Raf were involved in a pathway network connecting an extracellular signal to MAPK activation. ECM-involved proteins can be applied to sustain the 3D cell culture for dermal modeling developments in vitro. p38 MAP kinase was discovered to trigger mitosis mechanism in adult mammalian system, and our present work has been conducted to support the positive potential in human skin implant regenerations.

### 3.9. Secretions of Wound Healing Growth Factors on VEGF and TGF-*β*

For wound repair, the injury tissue requires many growth factors to stimulate hurt cell regeneration. VEGF and TGF-*β* are growth factors secreted by several cells, including platelets, macrophages, keratinocytes, fibroblasts, and ASCs [49]. Moreover, neutrophils, endothelial cells, and smooth muscle cells secrete VEGF. In this study, we tested secreted VEGF and TGF-*β* from ASC implants and injured zone tissues in Figures 6(c) and 6(d). VEGF concentrations in HA-L implants were too low to measure until seven days and compared to HA-H types were 12.23 ± 7.35 and 57.19 ± 4.10 pg/mL on day 7, and TGF-*β* amounts in HA-L and HA-H matrices were 36.81 ± 4.31 and 39.06 ± 2.03 pg/mL, respectively. A low ROS level helps cell proliferation as cell-cycle progression is activated and driven by growth factors. A review article by Gimble and Nuttall highlighted three categories of ASC secretions, including adipokines, cytokines, and secreted proteins [10]. Studies have pointed out that regulating HA affects the angiogenesis-related growth factors (e.g., VEGFA, VEGFB, hepatocyte growth factor (HGF), platelet-derived growth factor A (PDGFA), and PDGFC), causing enhanced tissue proliferation [49, 55]. VEGF is worth mentioning as a growth factor in dermal wound healing because it assists cell proliferation, migration, and angiogenesis. It is a major moderator in endothelial cell growth, proliferation, permeability, and migration of physiological and pathological angiogenesis. VEGF synergistically collaborates with other cytokines to enhance dermal damage tissue healing and to accelerate wound repair processes. ECM reconstructions are essential and secreted, synthesized, organized, and deposited via facilitations of VEGF around the wound surroundings. We discovered that VEGF secretion of cytokine response mediators in ASCs was greater in that HA-H matrix. We found that VEGF and TGF-*β* were upregulated in response to the ASC HA-H implant stimulus, and ECM was induced as well, possibly contributing to the upregulation the nutrient transcripts and above-mentioned protein interactions. Our implant generated an integrated response to ECM stiffness and TGF-*β*, a potent agonist of skin differentiation. The combination of ECM stiffness and exogenous TGF-*β* induced stem cell gene expressions more strongly than either worked via a p38 MAPK-dependent mechanism alone. This investigation has shown that autocrine VEGF and TGF-*β* pathway mechanisms were compensatory elements of ECM reconstructions in wound repair and were contingent upon the signaling in a feedback positive loop with p38, ERK, and NF-*κ*B.

Angiogenesis is the physiological process through which new blood vessels from preexisting vessels are formed in the earlier stage of vasculogenesis. Angiogenesis continues the growth of the vasculature by processes of sprouting and splitting. VEGF and TGF-*β* are presented to be major contributors for the angiogenesis to increase the number of capillaries in a given network. The capillary endothelial cells proliferate to show signs of tube structures upon stimulations by VEGF, TGF-*β*, and FGF-*β*. VEGF upregulation is a main factor of the physiological responses to exercise, and its role in angiogenesis is suspected to be a possible treatment in vascular injuries and skin damage. VEGF is an effective stimulator of angiogenesis, and in the presence of this growth factor, plated endothelial cells grow and migrate, eventually forming tube structures resembling capillaries. VEGF causes a massive signaling in endothelial cells and binds to VEGF receptor 2 to start a tyrosine kinase pathway which stimulates the production of factors to variously stimulate vessel permeability, proliferation/survival, migration, and finally differentiation into mature blood vessels. Mechanically, VEGF is upregulated with muscle contractions as a result of enlarged blood flow to affected areas. The amplified blood flow also causes a large increase in the mRNA productions of VEGF receptors 1 and 2.

**Figure 7:**
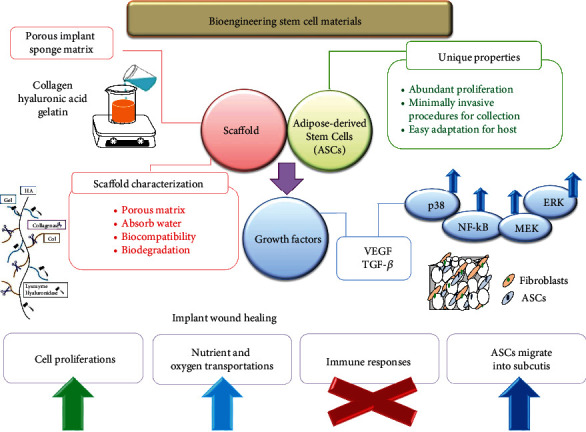
ASC HA-H porous biocompatible matrix cartoon chart for the wound repair.

The inflammation reaction is mainly involved in wound healing that is in early terms. If it is not properly adjusted, it will lead to abnormal wound healing. Based on our data, these HA fragments can stimulate ASCs to produce inflammation reaction cytokines, which transfer inflammatory signals to phagocytes and other dermis reaching the injury site. TGF-*β* is one of the most important cytokines involved in cellular growth, adhesion, migration, and ECM precipitation. TGF-*β* presents in the early terms of wound healing and adjusts ASC responses. A HA-rich surrounding contributed to the promotion of an inflammatory reaction during the wound healing course through activating proinflammatory phenotypes in ASCs [35]. HA modulates the proinflammatory cytokines to decrease oxidative stress and to enhance the skin wound repair [54]. The importance of inflammation mediators derived from the HA-H matrix-treated stem cells for activating the immune response was shown based on the activation of human blood leukocytes [36]. In injury repair procedures, TGF-*β* produced from similar cells compared to VEGF is essential in wound contraction, angiogenesis, cell penetration, connective tissue regeneration, inflammation, fibrotic scar formation, and reepithelialization [55]. TGF-*β*-treated ASCs induced higher expressions of type I Col along with cell cycle regulatory proteins and migration of fibroblasts. Considering the master cytokine leading to fibrosis, TGF-*β* supports defense of the normal tissue areas around wounded surroundings by forming granulations for the infections. It permits penetration of fibroblasts and the talk of fibroblasts to myofibroblasts, forming constrictive forces to help injury closures on ECM developments. To sum up, we prepared an ASC HA-H porous matrix for a promising implanted biomaterial in wound repair applications (Figure 7).

## 4. Conclusions

This study discovered that the HA-H matrix provided an optimal pore size and has a high water absorption level. The swelling ratios of both sponge matrices were analyzed by water absorption capabilities, and the results displayed that both have over 30-fold dry scaffold weight enhancements. In biodegradability experiments, two sponge implants could be degraded by three various human dermal enzymes. The ASC HA-H implant had an antioxidative ability to offer an increased cell proliferation rate and greater levels of VEGF and TGF-*β* amounts in related genes and proteins compared to other two groups and did not induce any immune responses *in vivo*. Overall, the study confirmed that the ASC HA-H implant can act as potent ECM for skin regeneration, and we recommend further investigations of the clinical properties be conducted on human skin wound healing.

## Data Availability

The data used to support the findings of this study are available from the corresponding authors upon request.

## Supplementary Materials

Supplementary Materials Table S1: wound repair-targeted sequences used in this study. Figure S1: *ex vivo* rat ASCs were cultured in HA-H matrix to evaluate by the fluorescent microscopy. The positive signals: CD90, CD105, and CD146; the negative signals: CD11b and CD31. Figure S2: western blot images.
