# Supplementary material for: Adipose-Derived Stem Cell-Incubated HA-Rich Sponge Matrix Implant Modulates Oxidative Stress to Enhance VEGF and TGF-β Secretions for Extracellular Matrix Reconstruction In Vivo
Source: Oxid Med Cell Longev. 2022 Jan 17;2022:9355692. doi: 10.1155/2022/9355692 (PMC8786469; doi:10.1155/2022/9355692)
Supplement: Supplementary Materials — Table S1: wound repair-targeted sequences used in this study. Figure S1: ex vivo rat ASCs were cultured in HA-H matrix to evaluate by the fluorescent microscopy. The positive signals: CD90, CD105, and CD146; the negative signals: CD11b and CD31. Figure S2: western blot images. [file 9355692.f1.doc]

Supplementary information

Adipose-derived stem cells incubated HA-rich sponge matrix implant modulates oxidative stress to enhance VEGF and TGF-β secretions for extracellular matrix reconstruction *in vivo*

Yu-Shen Cheng^1,†^, Hung-Hsun Yen^2,†^, Chung-Yen Chang^1^, Wei-Chih Lien^3^, Shu-Hung Huang^4,5^, Su-Shin Lee^4,5^, Peng-Yuan Wang^6^, Lin Wang ^7,*^, Hui-Min David Wang^5,8,9,*^

^1^ Department of Chemical and Materials Engineering, National Yunlin University of Science and Technology, Douliou, Yunlin 64002, Taiwan

^2^ Department of Fragrance and Cosmetic Science, Kaohsiung Medical University, Kaohsiung 807, Taiwan

^3^ Department of Physical Medicine and Rehabilitation, National Cheng Kung University Hospital, College of Medicine, National Cheng Kung University, Tainan, 701, Taiwan

^4^ Division of Plastic Surgery, Department of Surgery, Kaohsiung Medical University Hospital, Kaohsiung 807, Taiwan

^5^ Collage of medicine, Kaohsiung Medical University, Kaohsiung 807, Taiwan

^6^ Shenzhen Key Laboratory of Biomimetic Materials and Cellular Immunomodulation, Shenzhen Institute of Advanced Technology, Chinese Academy of Sciences, Shenzhen, Guangdong 518055, PR China

^7^ College of Chemistry & Pharmacy, Northwest A&F University, Yangling, Shaanxi 712100, PR China

^8^ Graduate Institute of Biomedical Engineering, National Chung Hsing University, Taichung 402, Taiwan

^9^ Department of Medical Laboratory Science and Biotechnology, China Medical University, Taichung 404, Taiwan

**Table S1.** Wound repair targeted sequences used in this study.

| P38 MAPK  forward primer, 5'-CAAGGGCAAGGACTACCTGG -3' reverse primer, 5'-TCTGGGGCAGGGACTGAATA -3'. |
| --- |
| NF-kB  forward primer, 5'-AAATCCGGGTCTTGTCCTGC-3' reverse primer, 5'-GCAAGCTGCCATTCTGTTCC-3''. |
| MEK-1  forward primer, 5'- ATT TGCCTGCATTACCGGTC-3' reverse primer, 5'- ATCAACGTTTTCTTTTCGG -3''. |
| MEK-2  forward primer, 5'- TGGGGAAGGTCAGCATTGC-3 reverse primer, 5'- GCCGCTCACCCCGAAGTCAC -3''. |
| ERK-1  forward primer, 5'- CCTGCTGGACCGGATGTTA-3' reverse primer, 5'- TGAGCCAGCGCTTCCTCTAC-3'. |
| ERK-2  forward primer, 5'- GGAGCAGTATTATGACCCAAGTGA-3' reverse primer, 5'- TCGTCCACTCCATGTCAAACT-3'. |
| β-Actin  forward primer, 5'-GCAGAAGGAGATCACAGCCCT-3' reverse primer, 5'-GCTGATCCACATCTGCTGGAA-3'. |

**Figure S1.** *Ex vivo* rat ASCs were cultured in HA-H matrix to evaluate by the fluorescent microscopy. The positive signals: CD90, CD105 and CD146; and the negative signals: CD11b and CD31.

**Figure S2.** Western blot images.
